# Signing and Verifying Encrypted Medical Images Using Double Random Phase Encryption

**DOI:** 10.3390/e24040538

**Published:** 2022-04-12

**Authors:** Hanaa A. Abdallah, Dalia H. ElKamchouchi

**Affiliations:** Department of Information Technology, College of Computer and Information Sciences, Princess Nourah Bint Abdulrahman University, P.O. Box 84428, Riyadh 11671, Saudi Arabia; haabdullah@pnu.edu.sa

**Keywords:** discrete wavelet transform, discrete cosine transform, double-random-phase encryption, digital signature, authentication, encryption

## Abstract

Digital Signature using Self-Image signing is introduced in this paper. This technique is used to verify the integrity and originality of images transmitted over insecure channels. In order to protect the user’s medical images from changing or modifying, the images must be signed. The proposed approach uses the Discrete Wavelet Transform to subdivide a picture into four bands and the Discrete Cosine Transform DCT is used to embed a mark from each sub-band to another sub-band of DWT according to a determined algorithm. To increase the security, the marked image is then encrypted using Double Random Phase Encryption before transmission over the communication channel. By verifying the presence of the mark, the authority of the sender is verified at the receiver. Authorized users’ scores should, in theory, always be higher than illegal users’ scores. If this is the case, a single threshold might be used to distinguish between authorized and unauthorized users by separating the two sets of scores. The results are compared to those obtained using an approach that does not employ DWT.

## 1. Introduction

Information security is to protect the integrity, confidentiality, and availability of data. Confidentiality is the process used to protect data from intruders who are not authorized to access the data. Authentication is the guarantee that both entities included in the communication can claim each other. Integrity assures that data are not modified or deleted by any unauthorized users and received data are exactly the same [1].

Electronic signatures obtained from content data are used to validate the identity of the signer of a document, similar to digital signatures. When a digital signature’s authentication mark is intentionally changed, the decoding of that signature yields content data that are completely different from the original data. Unlike digital signatures, however, digital watermark signatures have localization qualities that allow the precise location of probable content data changes to be determined. Furthermore, the goal of digital watermarks is to permanently and irreversibly label the image such that the attribution or assignment is unquestionable.

Digital watermarking inserts or hides information providing copyright protection intended for digital media. It has two major procedures, embedding and extraction. The procedures can be performed by means of hiding or visible methods. In order to protect possession and copyright, commonly digital watermarking has been applied in numerous purposes, for instance, content identification, authorization, and fingerprinting [2,3].

The following is the paper’s structure. Section 2 introduces a background of the Discrete Wavelet Transform (DWT), Discrete Cosine Transform (DCT), and the Double Random Phase Encryption, which is used in encryption. Section 3 produces the background review and related work. The proposed scheme is presented in Section 4. The experimental results are introduced in Section 5. Finally, Section 6 introduces the conclusion.

## 2. Background Review

### 2.1. Discrete Transforms

#### 2.1.1. Discrete Wavelet Transform (DWT)

**Figure 1:**
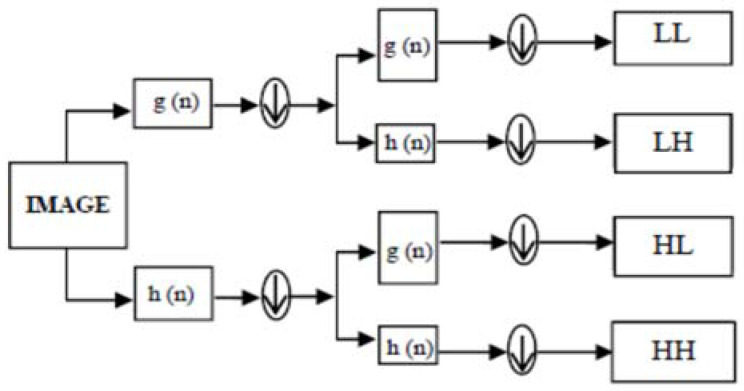
First-level discrete wavelet decomposition using filtering approach.

In [4], DWT is a mathematical tool which applies to decompose the images or frames into sub-bands. DWT is used in image watermarking, image compression, and removal of noise in audio and image [5]. DWT uses filters to split the signal into high- and low- frequency components. The edges are represented by high-frequency coefficients, which convey information about them, while the low-frequency coefficients are decomposed again into high- and low-frequency components. The wavelet transform is shown in Figure 1.

Where g (n) is the low pass filter, h (n) is the high pass filter, and down arrow (↓) is the down sampling of the output coefficients by factor two.

#### 2.1.2. Discrete Cosine Transform (DCT)

Discrete Cosine Transform is obtained from the real part of the Discrete Fourier Transform. It just carries the cosine term. The Discrete Cosine Transform (DCT) is transforming a signal from spatial domain to frequency domain. It is the result of combining cosine functions [6]. It divides an image into different frequency bands, to make embedding a watermark much easier. The middle frequencies are chosen to embed data they have minimized, which avoid the image’s most visually vital regions (low frequencies) without being overexposed to noise and compression attack (high frequencies) [7,8]. DCT domain watermarking can survive against the attacks, such as compression, sharpening, filtering, and noising.

The formal equations of DCT and IDCT as follows:

DCT equation:

(1) $$F\left(u,v\right)=\alpha \left(u\right)\alpha \left(v\right)\overset{N-1}{\underset{x=0}{\sum }}\overset{N-1}{\underset{y=0}{\sum }}f\left(x,y\right)\cos\frac{\left(2x+1\right)u\pi }{2N}\cos\frac{\left(2y+1\right)v\pi }{2N}$$

IDCT equation:

(2) $$F\left(x,y\right)=\overset{N-1}{\underset{x=0}{\sum }}\overset{N-1}{\underset{y=0}{\sum }}\alpha \left(u\right)\alpha \left(v\right)f\left(u,v\right)\cos\frac{\left(2x+1\right)u\pi }{2N}\cos\frac{\left(2y+1\right)v\pi }{2N}$$

where u,v,x,y=0, 1, 2, …,N−1, f (u, v) is the element of the transformed matrix, f (x, y) is the original matrix, and α(u) is defined as:

(3) $$\alpha \left(u\right)=\{\begin{array}{c} \sqrt{\frac{1}{N}}\;\;\;\;\;\;\;\;\;\;\;\;\;\;\;\;\;\;\;\;\;\;\;\;\;\;\;\;\;\;\;\;\;\;\;\mathrm{for}\;u=0\\ \sqrt{\frac{2}{N}}\;\;\;\;\;\;\;\;\;\;\;\;\;\;\;\;\;\;\;\;\;\;\;\;\;\;\;\;\;\;\;\;\;\mathrm{for}\;u\neq 0 \end{array}$$

### 2.2. The Double Random Phase Encryption (DRPE)

Encryption is the technique of transforming a message in a way that only authorized users can access it. DRPE was proposed by Refregier and Javidi, it is based on the modification of the spectral distribution of the image [9]. At the receiver, the user must know prior information about the modification or the target image in order to decode the image. The principle of DRPE is to encode an image to stationary white noise by using two independent random phase masks, and Fourier transforms in a coherent 4-f optical system. One key is inserted into the input plane (RPM1) to be encrypted, and the second key is inserted into the Fourier plane (RPM2).

Let *f* (*x*, *y*) be an input image where the positive *x* and *y* values denote the spatial domain coordinates. *f* (*x*, *y*) is modulated by a random phase  θ(x,y) given by

(4) $$\theta \left(x,y\right)=e^{i2\pi \theta_{0}\left(x,y\right)}$$

where $\theta_{0}\left(x,y\right)$ is a phase-function inserted into RPM1 and its value is randomly distributed over [0, 1].

We can obtain the Fourier transform of the modulated image after passing L1, the first lens. The second phase mask modulates the resulting image by the following equation:

(5) $$\emptyset \left(x,y\right)=e^{i2\pi \emptyset_{0}\left(u,v\right)}$$

where $\emptyset_{0}\left(u,v\right)$ is a phase-function inserted into RPM2and its value is randomly distributed over [0, 1].

The encoded image g(x,y) is resulting from the second lens L2, which performs the inverse Fourier transform by

(6) $$g\left(x,y\right)=FT^{-1\;}\left\{FT\left\{f\left(x,y\right)\cdot \theta \left(x,y\right)\right\}\cdot \varphi \left(u,v\right)\right\}$$

We can make the decryption procedure similar to the encryption procedure but in reverse order, using the equation

(7) $$f\left(x,y\right)=FT^{-1}\;\left\{FT\left\{g\left(x,y\right)e^{-i2\pi \emptyset_{0}\left(u,v\right)}e^{-i2\pi \theta_{0}\left(x,y\right)}\right\}\right\}$$

## 3. Related Work

In [10], Junling Zhang et al. introduced a new scheme where a signer creates a digital signature by applying a hash function on the message to create a mathematical message digest. The receiver can verify the sent message by applying the same hash function followed by the sender’s public key. Digital signatures can be verified by a trusted certification authority. In this study, an overview of digital signature and its potential applications in the Kingdom of Saudi Arabia were highlighted. It also provides a brief description about the establishment of a national certification authority to issue digital certificates, identifying the potential applications. To protect data privacy, Dang Hai et al. [11] proposed a message authentication code. The suggested mechanism allows for data integrity verification using only a portion of the original data. It was also shown to be secure against chosen-message-attacks and privacy-preserving. They also ran a test to see how much it cost to compute compared to a hash message authentication code. Sheryl et al. [12] developed a security system using hybrid of digital signature and DNA cryptography. The digital signature is applied using multi-feature biometric traits that include fingerprints and iris image. Then, they increased the security by embedding DNA cryptography on a smart card, and to prevent unauthorized access manually or digitally, they used geo-detection which compares the unregistered device’s location with the user’s location using any of their personal devices such as smart phone or table. In 2018 [13], Dubrova et al. presented a message authentication using secure cyclic redundancy check (CRC). It detects both random and malicious faults without raising bandwidth, according to the researchers, and the distinction from prior systems is that it uses random generator polynomials rather than irreducible generator polynomials. They offered a quantitative study of the attained security as a function of message and CRC sizes, demonstrating that the technique was appropriate for short messages. In 2018, Mohamed et al. [14] presented a hybrid security technique to secure the diagnostic text data in medical images. The model used either 2-D discrete wavelet transform 1 level (2D-DWT-1L) or 2-D discrete wavelet transform 2 level (2D-DWT-2L) steganography in addition to the encryption scheme. They proposed a hybrid encryption schema using a combination of Advanced Encryption Standard, Rivest, Shamir, and Adleman algorithms. This model begins by encrypting the secret data, then hiding the result in a cover image using 2D-DWT-1L or 2D-DWT-2L. In [15], Cui et al. introduced a secure energy-saving data aggregation scheme designed for large-scale WSNs. They applied a homomorphic encryption algorithm to protect end-to-end data confidentiality and used MAC to achieve in-network false data filtering and utilized the homomorphic MAC algorithm to achieve end-to-end data integrity. In 2020 [16], Abeer et al. proposed a novel cryptosystem that consists of three stages, namely, fusion, substitution, and chaotic permutation, to apply confidentiality and secrecy. The first stage is the DWT fusion with the averaging fusion rule. The second stage supports the substitution through either DCT or DST. Finally, the third stage utilizes chaotic encryption to apply confusion to the signal. In the same year, for the protection of medical data, Anand et al. [17] introduced an upgraded DWT–SVD domain watermarking. To reduce the noise distortion of the text watermark, hamming coding is used. They examined two different encryption algorithms and three different compression schemes and found Chaotic-LZW (Lempel–Ziv–Welch) to be the most efficient. In [18], Nadir et al. presented an innovative procedure designed for embedding a ciphered electronic record for a patient by means of the Discrete Cosine Transform (DCT), where the medical image resultant is decomposed by means of the Discrete Wavelet Transform (DWT), then, the electronic record of the patient will be ciphered by means of the Elgamal cryptosystem, subsequently applying the Arnold map in order to increase the levels of unpredictability and randomness, which increase the system complexity. Recently, in 2022, ref. [19] S. Prasanth Vaidya presented a novel adaptive watermarking system utilizing a hybrid transform and patient fingerprint as a watermark intended for electronic health care techniques. The proposed algorithm shows its robustness and authentication with numerous medical image assaults. Additionally, in [20], a watermarking approach for patient identification and watermark integrity verification was presented, where a discrete wavelet transform is employed to divide the medical image into four sub-bands used for the integration procedure. Most recently [21], in 2022, Mohammad et al. introduced a BioHashing and watermarking method for ensuring the integrity, validity, and secrecy of various medical photographs. The BioHashing approach is utilized to generate the watermark on the sender side, all the conditions for the robustness and imperceptibility are met by the suggested technique.

## 4. Discussion

### 4.1. Signing Process

**Figure 2:**
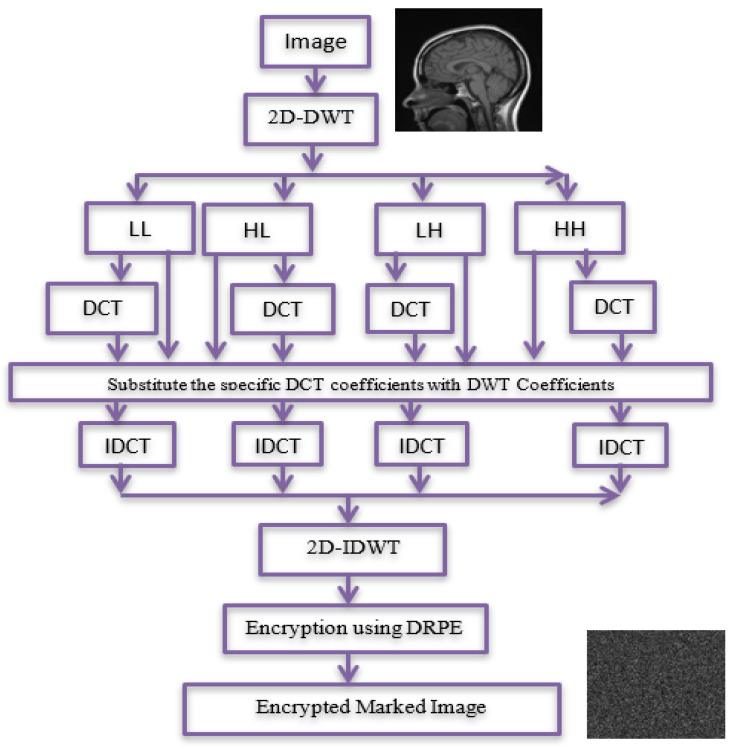
Signing process.

The signing process introduces the stages of signing and encrypting the data as shown in Figure 2. The medical image is applied as a plain image, and the cipher image will be a signed image by using the block content-based technique. The marked image is then encrypted to increase the level of security. The proposed technique is applied on the DWT sub-bands of the image. The signing process is described below:

Stage 1: Compute the 2D-DWT of the original medical image A. This operation generates four quarters LL, HL, LH, and HH. Each sub-band is a matrix of DWT coefficients at a specific resolution. Then, the four sub-bands are divided into (8 × 8) blocks.

Stage 2: For each sub-band, permutation is done by replacing coefficients from right to left, bottom to top through DCT coefficients of (LL, HL, LH, and HH) sub-bands.

Stage 3: For the first row and first column of each DCT, the coefficients for each block in the first sub-band (LL) are substituted by the last row and last column, respectively, of a corresponding block in the second sub-band (HL). For first row and column of each DCT, coefficients of each block in the third sub-band (LH) are substituted by the last row and column of a corresponding block in the fourth sub-band (HH). Then, IDCT and 2D-IDWT are applied.

Stage 4: The signed image is encrypted using DRPE encryption technique.

### 4.2. Verification Process

**Figure 3:**
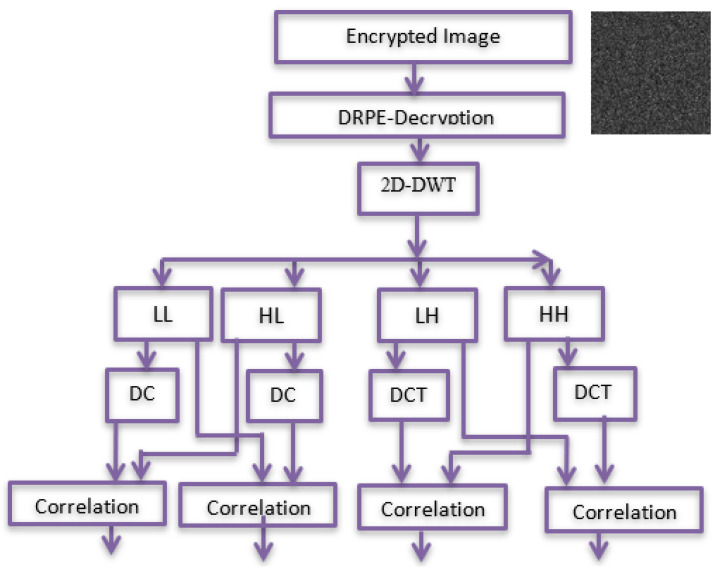
Verification process.

The encrypted marked image is decrypted to reconstruct the marked image as shown in Figure 3. Then, the marked one is verified. This is shown in the following stages:

Stage 1: The encrypted marked image is decrypted using DRPE to obtain a marked image.

Stage 2: 2D-DWT is applied to the marked image to generate four sub-bands LL, HL, LH, and HH. Four sub-bands are divided into (8 × 8) blocks; then, DCT is applied to each block.

Stage 3: Working from right to left, bottom to top, embedded rows and columns are extracted and compared to their corresponding cones through a correlation analysis.

Stage 4: Embedded rows and columns are extracted and compared to their corresponding cones through a correlation analysis.

## 5. Simulation Results and Discussions

For performance evaluation of the proposed frameworks, simulation tests are introduced to assess the efficiency of the proposed approaches. All proposed encryption approaches are implemented with MATLAB (R2017a). Different metrics and perspectives have been considered in the performance evaluation process. The proposed technique has been applied to a medical image and the results are shown in Table 1. The correlation coefficient between extracted rows or columns and the original ones is used to measure the signature’s resilience, while the peak signal-to-noise ratio (PSNR) is used to determine the image quality after marking or embedding, as well as encryption and decryption. The higher the PSNR, the better the signing and encryption algorithm. Higher PSNR means a higher imperceptibility [22]. This measure is given mathematically by the following equation:

(8) $$PSNR\;(dB)=10\log_{10}\left(\frac{255^{2}}{\frac{1}{N^{2}}\sum_{x,y}{\left(A_{w}(x,y)-A(x,y)\right)}^{2}}\right)$$

where *A* (*x*, *y*) is the original image and *A_w_* (*x*, *y*) is the signed image.

The Correlation Coefficient measure is used between the extracted coefficients and the original coefficients [23], this measure is given mathematically by

(9) $$c_{r}\left(W,\hat{W}\right)=\frac{\sum_{y}W(y)\hat{W}(y)}{\sqrt{\sum_{y}W^{2}(y)\sum_{y}{\hat{W}}^{2}(y)}}$$

where W and $\hat{W}$ are the original and extracted marks, correspondingly.

### 5.1. Structural Similarity Index (SSIM)

The structural similarity index is a measure of how similar two structures are (SSIM). The SSIM is used to determine whether the plain signal and the encrypted or marked signal are similar. It provides a more accurate assessment than the Mean Square Error (MSE). Equation (10) [22] is used to compute it.

(10) $$\mathrm{SSIM}\left(x,y\right)=\frac{\left(2\mu_{x}\mu_{y}+C_{1}\right)\left(2\sigma_{xy}+C_{2}\right)}{\left(\mu_{x}{}^{2}+\mu_{y}{}^{2}+C_{1})(\sigma_{x}{}^{2}+\sigma_{y}{}^{2}+C_{2}\right)}$$

where *μ_x_* and *μ_y_* represent the mean values for signals *x* and *y*, correspondingly, *σ_x_*, and *σ_y_* are the variances of the signals *x* and *y*, respectively, *σ_xy_* is the cross-covariance between two signals *x* and *y*. *C*_1_ and *C*_2_ have small values, the SSIM value is less than one. The correlation coefficient between extracted rows or columns and the original ones is used to assess the mark or signature’s robustness, while the mean square error (MSE) and peak signal-to-noise ratio (PSNR) are used to assess the image quality after marking or embedding.

Table 1 shows the original image, marked image, encrypted image, the decrypted image, and the correlation between extracted blocks and original blocks after decryption using the Double Random Phase Encryption. It is clear that the encryption increases the security, and the mean correlation between the recovered mark and the original mark is measured to be 0.9 in both cases.

The PSNR and SSIM between the original image and marked image are 35.99 db and 0.97, respectively, which means that the marking does not affect the quality of the original image and keeps the invisibility of mark in the image. After encrypting the marked image using the DPRE algorithm, the results show that PSNR and SSIM between the encrypted and marked image are 12 db and 0.1, which verifies the strength of the suggested cryptosystem, as there is no similarity between them. Moreover, the results show that the correlation between the embedded rows and columns with the original is near to 1, verifying the robustness of the mark or signature.

**Figure 4:**
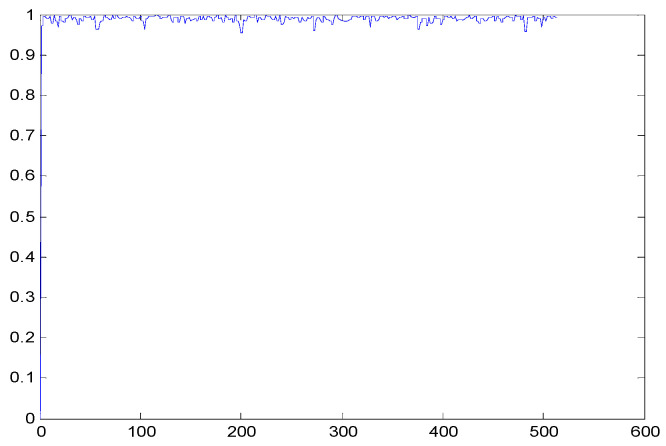
Block-based correlation.

The study of the effect of noise on the encrypted and marked image in addition to the effect of noise on the recovered mark is shown in Table 2. It is clear that by increasing the additive white Gaussian noise (AWGN), the SNR between the noisy image and original image takes values from 5 dB to 50 dB, the correlation between the original and recovered rows and column with original, i.e., the correlation between extracted blocks and original blocks increased from 0.1 to 0.75, which verifies that the recovered mark does not match the original mark. The correlation values which are used to express the similarity between blocks in a marked image and blocks in a non-marked image are calculated in Table 3. A higher correlation means that the image is marked, this is explained as the blocks which have a mark within row or columns get a high value of correlation, which is near 1, but the others with no mark get low values of correlation. Correlation values in Figure 4 ensure the possibility of mark verification, which means that we can verify the presence of a mark when the correlation between the recovered rows and columns and the original is equal to 1, as it illustrates the correlation between blocks of a marked image which equals to 1. As a result, this confirms that the image is marked. In Table 3, we did not apply marking to the image, and we divided the image by the same technique and compared the blocks with each other. We find that the correlation is close to zero and this means that the image is not marked by this method. As a result, we verify the originality of the image which contains the mark.

From Table 4, it has been noticed that the correlation of the original image is high (CF = 0.9143) and close to one. In contrast, the correlation of the encrypted image is minimal (CF = 0.0071) and close to zero, which demonstrates that encryption using the DRPE algorithm can break the relativity efficiently, possesses a robust capability to withstand a statistical attack, and does not alter the quality of the output decrypted image after inserting the mark, i.e., the decrypted image is the same as the original.

### 5.2. Comparative Analysis

The proposed scheme is compared with other existing schemes using DWT and DCT transformations in their designs in order to make a generic comparison based on a set of generic performance indicators. Table 5. shows that by applying Gaussian noise to the correlation of the mark with the original mark, the proposed technique offers a better value, indicating the robustness of the proposed technique. Table 6. further compares our proposed scheme with other schemes, which proves that our suggested scheme is able to avoid an AWGN attack and achieve a favorable trade-off between resistance and imperceptibility due to its embedding method and additive Gaussian noise attack.

Table 6 shows the comparative study with the techniques which use watermark as a mark. We found that our technique, which is different in methodology with these techniques, presents a high correlation when comparing to the techniques in [26]. It is shown that when we apply (AWGN) noise to a marked image and we get the correlation between the recovered mark and original, we find that the correlation is higher than that of the techniques using DWT, SVD, DWT-based SVD and DT-CWT-based SVD, as our technique gives a correlation between 0.1 to 0.75 and these techniques give a correlation in the range from 0.05 to 0.3 which means that our technique is more robust. Figure 4 shows that the correlation between the intended rows and columns are close to 1, which means the image is marked.

**Figure 5:**
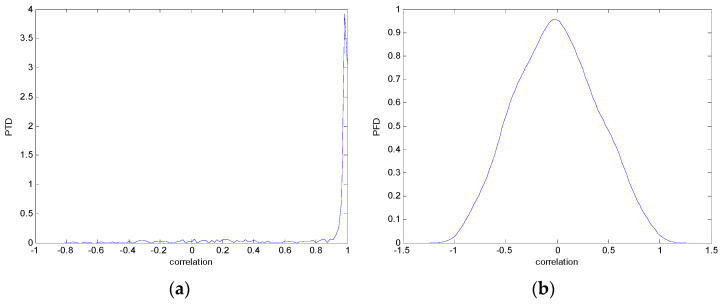
PTD and PFD using digital signature (**a**) PTD and (**b**) PFD.

The test data for picture authentication systems include both marked and unmarked images. The Probability of True Distribution (PTD) and Probability of False Distribution (PFD) are used to assess the correlation scores obtained during the authentication stage. As illustrated in Figure 5a, the Probability of True Distribution (PTD) is the probability distribution of the correlation between the designated image blocks, the marked blocks. The Probability of False Distribution (PFD) is the probability distribution of the correlation between the blocks of the unmarked image as shown in Figure 5b.

## 6. Conclusions

This paper investigates image integrity and privacy by using a digital signature and an encryption algorithm. The results showed that the proposed scheme has the ability to verify the presence of the mark as the embedded rows and columns are extracted and compared to the original cones through the correlation analysis. Moreover, after the addition of the AWGN noise to the marked image, the result showed that we still can verify the presence of the mark; therefore, it can resist different attacks. The proposed technique can be used to ensure the originality of data transmitted via channels and detect any odd modifications to the designated image. The upcoming exploration focus is primarily to utilize machine learning as well as pattern recognition system intended for better performance optimization and using new frequency domain transforms and singular value decomposition to resist various attacks.

## Figures and Tables

**Table 1:** Results of marking and encrypting a medical image.

**Original Image**	**Marked Image**	**Encrypted Image (Chaotic)**	**Decrypted Image**	**Correlation between Extracted Blocks and Original Blocks**
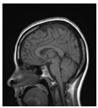	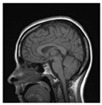 **PSNR = 35.99 dB** **SSIM = 0.97**	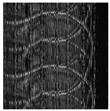 **PSNR = 12.17 dB** **SSIM = 0.1035**	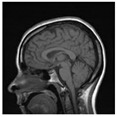 **PSNR = 35.99 dB** **SSIM = 0.97**	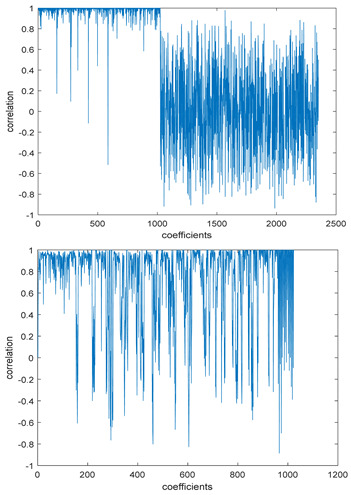
**Original Image**	**Marked Image**	**Encrypted Image (DRPE)**	**Decrypted Image**	**Correlation between Extracted Blocks and Original Blocks**
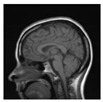	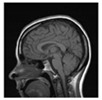 **PSNR = 34.26 dB** **SSIM = 0.96**	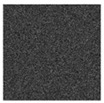 **PSNR = 12.84 dB** **SSIM = 0.0349**	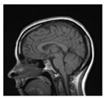 **PSNR = 34.37 dB** **SSIM = 0.96**	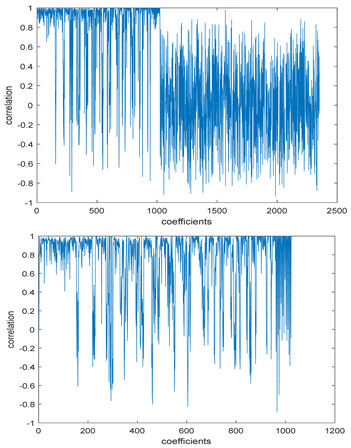

**Table 2:** The effect of AWGN noise on DRPE-encrypted images.

AWGN (SNR)	Correlation between Extracted blocks and Original Blocks
**5 dB**	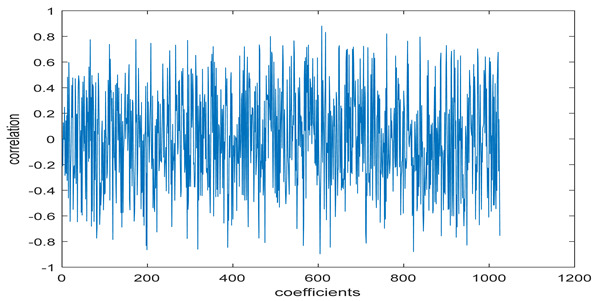
	**Mean correlation = 0.1192**
	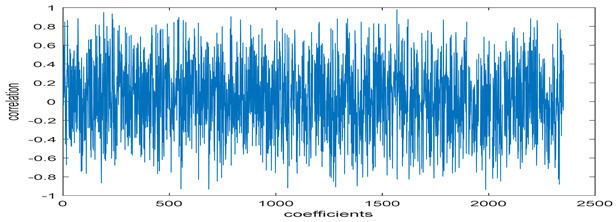
	**Mean correlation = 0.0374**
**10 dB**	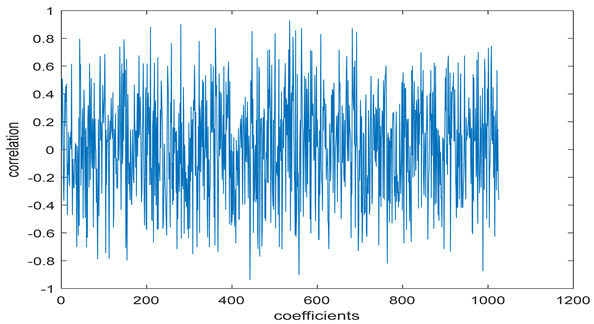
	**Mean Correlation = 0.4416**
	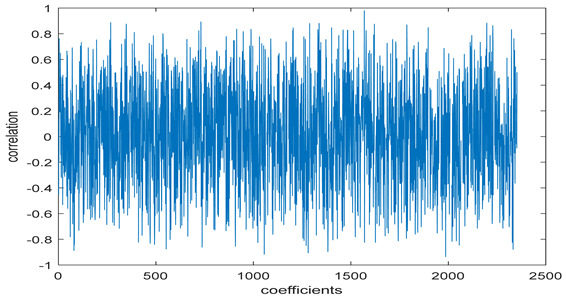
	**Mean Correlation = 0.0737**
**20 dB**	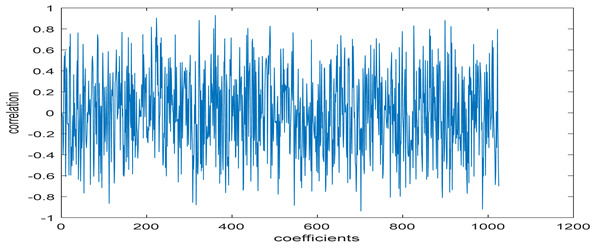
	**Mean Correlation = 0.3597**
	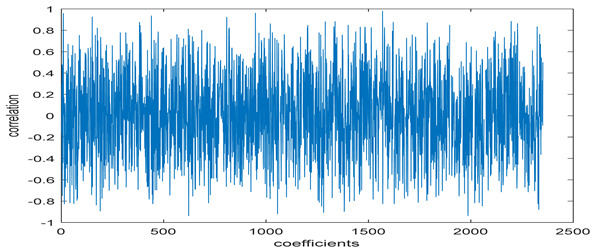
	**Mean Correlation = 0.1600**
**30 db**	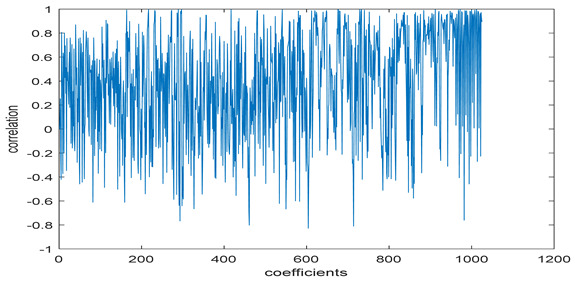
	**Mean correlation = 0.5407**
	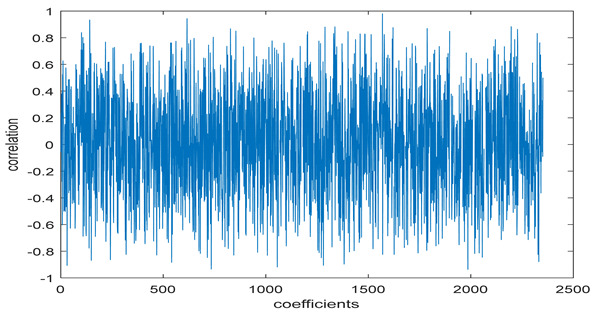
	**Mean correlation = 0.3117**
**50 dB**	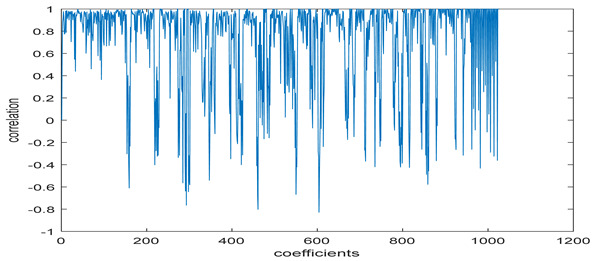
	**Mean correlation = 0.7592**
	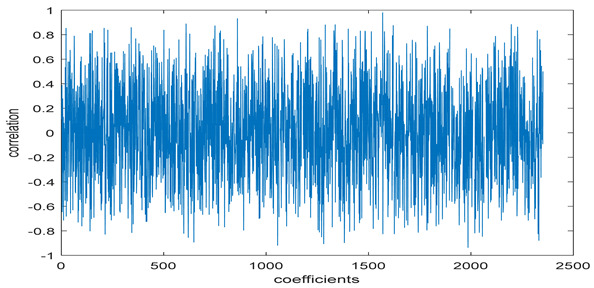
	**Mean correlation = 0.6159**

**Table 3:** Correlation between the original image and the marked image.

Original Image	Original Image with No Mark	Correlation between Extracted Blocks and Original Blocks in Image with No Mark
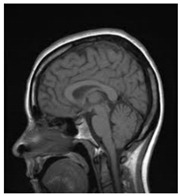	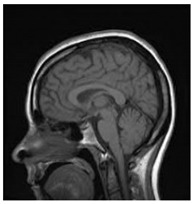	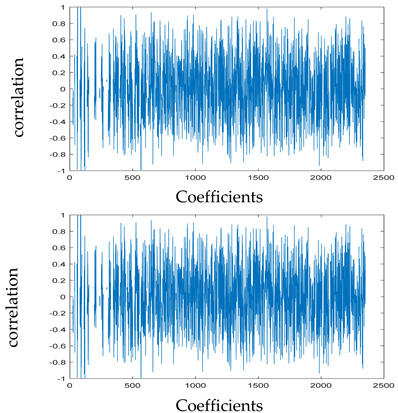

**Table 4:** Horizontal correlation between the original image and the encrypted image.

Original Image	Original Image with Mark
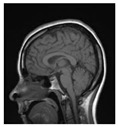	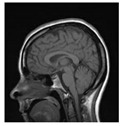
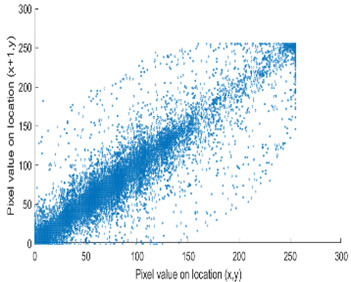	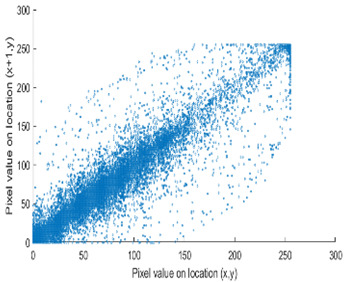
**Encrypted Image**	**Decrypted Image**
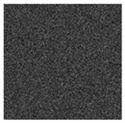	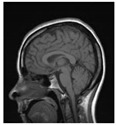
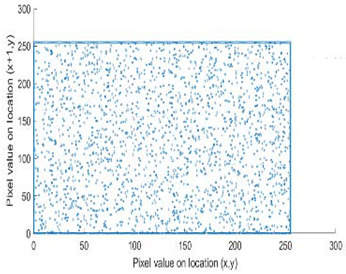	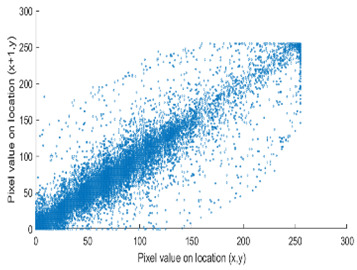

**Table 5:** Comparative study with other techniques.

Gaussian Noise	CR (Correlation)			
	Proposed Scheme	[24]	[25]	[26]
Gaussian noise 0.5	0.75	0.63	0.58	0.28

**Table 6:** Comparative study with other techniques of marking [26].

Correlation (cr)									
	DWT		SVD	DWT Based SVD		DT-CWT Based SVD			
	cr1	cr2	cr1	cr1	cr2	cr1	cr2	cr3	cr4
No attack	0.95	0.96	0.99	0.99	0.98	0.9996	0.9996	0.9996	0.9997
Rotation angel									
−90	0.09	0.1	0.99	0.34	0.25	0.8253	0.9551	0.8527	0.9763
−1	0.12	0.1	0.44	0.65	0.71	0.9655	0.9340	0.9898	0.9655
Noise variance									
0.1	0.05	0.06	0.04	0.13	0.18	0.3129	0.3353	0.3613	0.3926
0.5	0.03	0.04	0.002	0.1	0.14	0.2242	0.2393	0.2486	0.2703
Resizing ratio									
0.7	0.21	0.22	0.74	0.72	0.9	0.9766	0.9740	0.9866	0.9896
0.9	0.35	0.35	0.95	0.94	0.97	0.9873	0.9956	0.9960	0.9973
Compression quality									
10%	0.3	0.26	0.81	0.97	0.97	0.9875	0.9906	0.9931	0.9937
30%	0.55	0.48	0.93	0.97	0.98	0.9992	0.9978	0.9991	0.9985
Cropping ratio									
512–300	0.23	0.16	−0.73	0.38	0.22	0.7600	0.6567	0.87	0.7719
512–100	0.19	0.13	−0.87	0.21	0.09	0.6317	0.5882	0.6970	0.7139

## Data Availability

Not applicable.
